# Strong long‐range temporal correlations of beta/gamma oscillations are associated with poor sustained visual attention performance

**DOI:** 10.1111/ejn.13672

**Published:** 2017-10-06

**Authors:** Mona Irrmischer, Simon‐Shlomo Poil, Huibert D. Mansvelder, Francesca Sangiuliano Intra, Klaus Linkenkaer‐Hansen

**Affiliations:** ^1^ Department of Integrative Neurophysiology Center for Neurogenomics and Cognitive Research (CNCR) Amsterdam Neuroscience VU University Amsterdam 1081 HV Amsterdam The Netherlands; ^2^ NBT Analytics BV Amsterdam The Netherlands; ^3^ IRCCS Don Gnocchi Foundation Milan Italy

**Keywords:** alpha, DFA, LRTC, neuronal oscillations, performance

## Abstract

Neuronal oscillations exhibit complex amplitude fluctuations with autocorrelations that persist over thousands of oscillatory cycles. Such long‐range temporal correlations (LRTC) are thought to reflect neuronal systems poised near a critical state, which would render them capable of quick reorganization and responsive to changing processing demands. When we concentrate, however, the influence of internal and external sources of distraction is better reduced, suggesting that neuronal systems involved with sustained attention could benefit from a shift toward the less volatile sub‐critical state. To test these ideas, we recorded electroencephalography (EEG) from healthy volunteers during eyes‐closed rest and during a sustained attention task requiring a speeded response to images deviating in their presentation duration. We show that for oscillations recorded during rest, high levels of alpha‐band LRTC in the sensorimotor region predicted good reaction‐time performance in the attention task. During task execution, however, fast reaction times were associated with high‐amplitude beta and gamma oscillations with low LRTC. Finally, we show that reduced LRTC during the attention task compared to the rest condition correlates with better performance, while increased LRTC of oscillations from rest to attention is associated with reduced performance. To our knowledge, this is the first empirical evidence that ‘resting‐state criticality’ of neuronal networks predicts swift behavioral responses in a sensorimotor task, and that steady attentive processing of visual stimuli requires brain dynamics with suppressed temporal complexity.

## Introduction

Research on attention has a long tradition in investigating how we selectively process one object while ignoring others (Driver, 2001). In the face of limited processing abilities, task performance is strongly influenced by the level of attention paid to the task (Kahneman, 1973 in: (Sarter *et al*., 2006)). Still, competing influences such as additional sensory information (Theeuwes, 2010) or mind wandering (Smallwood & Schooler, 2015) can cause distraction from the task at hand and dramatically reduce performance.

Cognitive functions like mind wandering evolve across many time scales, from brief mental images to long‐lasting trains of thoughts (Buckner *et al*., 2008; Seli *et al*., 2014). The underlying mechanism therefore also needs to be capable of organizing the coordination of neuronal activity on many time scales (Linkenkaer‐Hansen *et al*., 2005). Indeed, both hemodynamic (Fox *et al*., 2006) and electrophysiological measures (Linkenkaer‐Hansen *et al*., 2001; Montez *et al*., 2009; Palva *et al*., 2005) indicate that ongoing brain activity is highly variable and shows organization on many time scales. Quantitatively, this coordination is reflected in long‐range temporal correlations (LRTC) of the type observed in non‐linear dynamical systems operating near the critical state (Chialvo, 2010; Kello *et al*., 2010; Poil *et al*., 2012). This is indicating that ongoing brain activity harbors a long‐term memory process known as 1/*f* noise (Gilden, 2001), a hallmark of healthy physiological systems with high demands for swift adaptation such as heartbeat (Goldberger *et al*., 2002) and gait (Hausdorff *et al*., 1996), as well as the ability to produce repetitive taps using the index finger (Torre *et al*., 2011) and threshold‐stimulus detection tasks (Palva *et al*., 2013). Deviations from the normal range of LRTC have been associated with diseases such as clinical depression (Linkenkaer‐Hansen *et al*., 2005), Alzheimer (Montez *et al*., 2009), epilepsy (Monto *et al*., 2007), Parkinson's (Hohlefeld *et al*., 2012), and autism (Lai *et al*., 2010). Therefore, associating the success of overt behavior to oscillatory brain dynamics can be valuable for understanding general functioning of the brain and may have prognostic properties for optimal functioning or disease.

LRTC have been suggested to reflect the degree to which the brain remains capable of quick reorganization (Deco *et al*., 2013; Linkenkaer‐Hansen *et al*., 2001; Singer, 2013; Tognoli & Kelso, 2014) and, thus, is responsive to different processing demands. The closer neuronal systems are to the critical point separating the ordered sub‐critical and the disordered super‐critical regime, the stronger the long‐range temporal correlations in activity fluctuations (Poil *et al*., 2012). The transition from resting state to attention‐task activity on the other hand shows a decrease in the long‐range memory of the signal as found during focused attention meditation in experienced meditators (Irrmischer *et al*., 2017), as well as during a cued response task in electroencephalography (EEG) (He *et al*., 2010) and in fMRI BOLD activations (He, 2011); however, large fluctuations in ongoing oscillations remain during task execution and these have been associated with trial‐by‐trial variability in performance (Linkenkaer‐Hansen *et al*., 2004b; He & Zempel, 2013). Still, it remains unknown whether this change in temporal structure of continuous amplitude modulations of oscillatory activity is a mere by‐product of the shift from idling resting state to task engagement or whether it is behaviorally relevant in terms of performance success. And, more specifically, whether brain states closer to the critical regime—previously associated with high adaptability and versatile information processing (Bak, 1996; Kinouchi & Copelli, 2006; Shew *et al*., 2009)—is beneficial for a sustained attention task, or whether a state characterized by less complex variability is better?

In this study, based on earlier findings of reduced LRTC during focused attention meditation (Irrmischer *et al*., 2017), we propose the working hypothesis that the human brain is poised near a critical state that makes attention inherently unstable and, consequently, a less volatile brain state is desired when sustained focus of attention is required. A key prediction derived from this hypothesis is that long‐range temporal correlations in human EEG oscillations are suppressed during sustained attention and that such suppression may be related to behavioral performance. To test this, we analyzed the changes in LRTC from rest to active state, and their correlation to performance in a sustained attention task, which due to its repetitive nature and length was specifically targeted at inducing distractions in the form of mind‐wandering.

## Methods

### Participants

The 57 participants were healthy students of the Vrije Universiteit in Amsterdam, and volunteers from the general population aged 20–48 years (M = 25 years, SD = 6.2; 35 females), with no history of neurological complications including ADHD, depression, or substance abuse. All participants signed the informed consent, and the protocol was approved by the Scientific and Ethical Review Board (VCWE) of the Faculty of Psychology and Education, VU University Amsterdam.

### EEG measurements

EEG recordings were acquired using the Electrical Geodesics EEG system (GES200) with 128‐EGI HydroCel channel sponge‐based EEG‐caps at a sampling rate of 1000 Hz and using Cz as reference electrode. Participants were measured while sitting alone in the recording room in front of a computer screen. The experiment was programmed in OpenSesame (Mathôt *et al*., 2012).

### Experimental conditions

#### Eyes‐closed rest

Participants were first measured during 5‐min eyes‐closed rest (ECR) while sitting on a chair. The instruction was ‘Please keep your eyes closed, relax, and try not to fall asleep’.

#### Continuous temporal expectancy task

**Figure 1:**
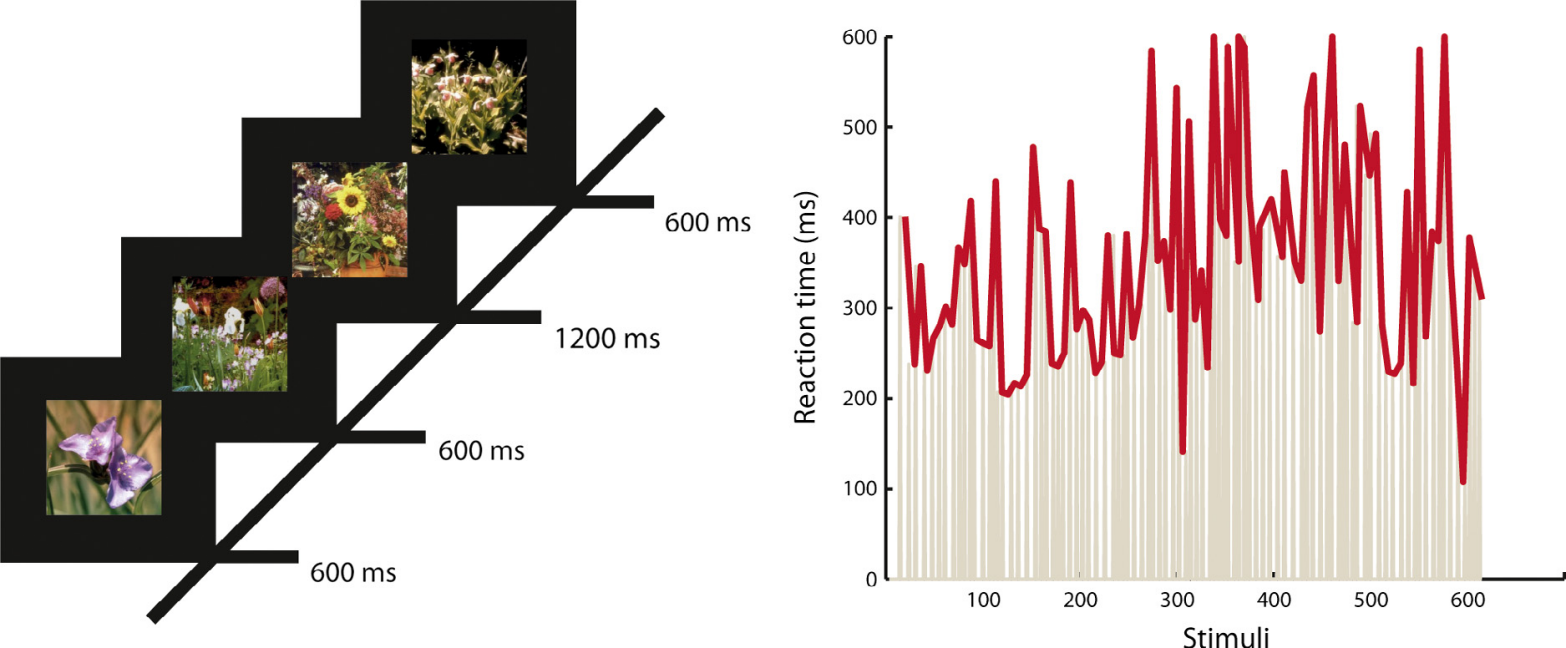
The Continuous Temporal Expectancy Task (CTET). An adaptation of the original task (O'Connell *et al*., 2009) was used which consisted of centrally presented photographs of flowers shown at regular intervals (600 ms) and longer intervals (1200 ms), which occurred semi‐randomly (every 4th–10th stimuli) 100 times. The entire task took 7.5 min, and participants were asked to press the space bar with their right hand when a stimulus was presented longer.

Next, participants completed a sustained attention task (adaptation of CTET (O'Connell *et al*., 2009)), which was designed to measure lapses in attention through the number and timing of errors the participants make. The task consisted of centrally presented photographs of flowers shown at regular intervals (600 ms), resulting in a continuous stream of pictures. Participants were asked to attend to the temporal duration of each stimulus and press the space bar with their right hand when a stimulus was presented longer (1200 ms) than the standard duration (Fig. 1). Long‐duration stimuli occurred semi‐randomly (every 4th–10th stimuli) 100 times. Identifying the duration target is easy when fully attending to the stimuli; however, it quickly becomes demanding to continuously focus on the boring task resulting in great variation as well as occasional misses during the 7.5‐min task. This makes the continuous temporal expectancy task (CTET) (O'Connell *et al*., 2009) a measure of continuous deployment of attention to the time domain, that is, duration of repetitive events. The stimuli were made of naturalistic pictures taken from the International Affective Picture System (IAPS; (Lang *et al*., 1999)), with pictures specifically chosen for their low arousal values. Additionally, the color brightness, saturation, and size of the scenes were standardized to decrease stimulus perception‐dependent differences.

#### Eyes‐open rest

In 23 subjects, we also recorded a 5‐min eyes‐open rest (EOR) condition with the instruction ‘Please keep your eyes open, relax, focus on the mark on the screen and try not to fall asleep’. This was performed to assess whether differences in brain dynamics between the resting state and the CTET experiment were solely due to having eyes open or not.

### Rationale

The capability to monitor the duration of stimuli is increased when attention is actively oriented toward it (Nobre *et al*., 2007) and decreases if top‐down attentional effort is diminishing. Lapses in identifying targets are therefore a direct correlate of decreased attention, a phenomenon seen in many everyday life applications. Different from conceptually similar attention tasks such as the go/no‐go ‘Sustained attention to response task’ (SART; (Robertson *et al*., 1997)), every stimulus is a potential target and only discriminated by its longer presentation time and not by perceptual features. This solves the problematic issue of target salience and automatically engaged exogenous attention interfering with the continuous attentional aspect which is targeted in this study (Robertson & O'Connell, 2012). Finally, it has been shown that performance declines after just 3 min of task performance, but there is no broader decline over blocks indicating that the CTET is a useful paradigm for tracing drifts in the level of attention over time (O'Connell *et al*., 2009). Therefore, in this study, a single block of 7.5 continuous minutes was chosen to tap into long‐term sustained attention abilities of the participants.

### EEG pre‐processing

The EEG signals were FIR‐filtered (0.5–45 Hz band‐pass, Blackman window with 1 Hz transition band), and noisy channels were removed using the pre‐processing functions available in EEGLAB and the Neurophysiological Biomarker Toolbox (NBT, http://www.nbtwiki.net) (Hardstone *et al*., 2012). All signals were visually inspected in windows of 10 s and transient artifacts, for example, caused by head movements or eye blinks were manually marked and omitted from the subsequent computations of spectral power and detrended fluctuation analysis (DFA). Typically, only 1–2 s around an artifact were marked. Subsequently, we re‐referenced the signals to the common average and applied Independent Components Analysis (Infomax) (Bell & Sejnowski, 1995; Makeig *et al*. 2000) for identification of independent components related to heartbeat or eye movements, which were visually identified and removed (on average only two components were removed). The remaining components were projected back to signal space.

### Behavioral and EEG analysis

The observed reaction‐time averages were calculated from the point in time when the target stimulus was displayed longer than non‐target stimuli. The reaction time therefore includes both the time needed to notice the deviant and the time to react. The next stimulus is displayed 600 ms after and to prevent that wrong presses to non‐target stimuli would count as a very slow reaction to the target stimulus, we defined the maximum allowed reaction time to 900 ms. To avoid short reaction times in subjects responding very fast but also missing several trials, and to obtain a comprehensive performance measure that also included the misses and too slow responses, we defined misses to have the longest reaction time allowed (i.e., 900 ms). We note that reaction times and number of errors exhibited very similar associations with LRTC of neuronal oscillations during CTET and, therefore, we only report the results for the reaction‐time measure defined above.

For the EEG analysis, we computed the power in the five classical frequency bands (delta 1–4 Hz, theta 4–8 Hz, alpha 8–13 Hz, beta 13–30 Hz, and gamma 30–45 Hz) using the Welch method with a 4096‐point Hamming window and a frequency resolution of 0.25 Hz. The relative power was calculated by dividing the absolute power in each frequency band with the integrated power in the range 1–45 Hz. The EEG analysis was performed per channel with a non‐directional *t*‐test (significance level: *P *<* *0.05), and, due to the continuous nature of the reaction times, the parametric Pearson's correlation coefficient was used to test for correlations with reaction times (significance level: *P *<* *0.05). To prevent chance‐level effects, we used the binomial multiple‐comparison correction method, which tests whether a significant number of channels reach the significance level of *P *<* *0.05 within a specific frequency band. The likelihood of having 12 channels of 128 by chance is <2% (cf. binomial distribution) (Montez *et al*., 2009; Nikulin *et al*., 2012; Schiavone *et al*., 2014). All exponents reported in the main text are averages of significant electrodes across subjects, ± SEM.

### Quantifying long‐range temporal correlations

To quantify the strength of LRTC in the amplitude modulation of the EEG oscillations, we first extracted the amplitude envelope of each frequency band using band‐pass filters (FIR‐filter, Blackman window with transition bandwidth of 1 Hz) and the Hilbert transform. Next, the root‐mean‐square fluctuation of the integrated and linearly detrended signals, *F*(*t*), was calculated as a function of time window size, *t* (with an overlap of 50% between windows) and plotted in double‐logarithmic coordinates. The DFA exponent is the slope of the fluctuation function *F*(*t*) in a given interval, which was set to 5–30 s for delta and theta band, from 2–30 s for alpha and 1–30 s for the beta and gamma bands. The lower time scale of fitting the power law is higher for the slow oscillations in order to exclude the temporal autocorrelations introduced by the band‐pass filters. A DFA exponent α = 0.5 indicates randomly fluctuating oscillation amplitudes (no temporal structure), whereas 0.5 < α < 1.0 indicates LRTC with the temporal inhomogeneity of fluctuations increasing with increasing DFA exponents. The main steps from the broadband signal to the quantification of LRTC using DFA have been described in detail previously (Hardstone *et al*., 2012; Linkenkaer‐Hansen *et al*., 2001).

## Results

### Sustained attention is associated with a reduction in LRTC

**Figure 2:**
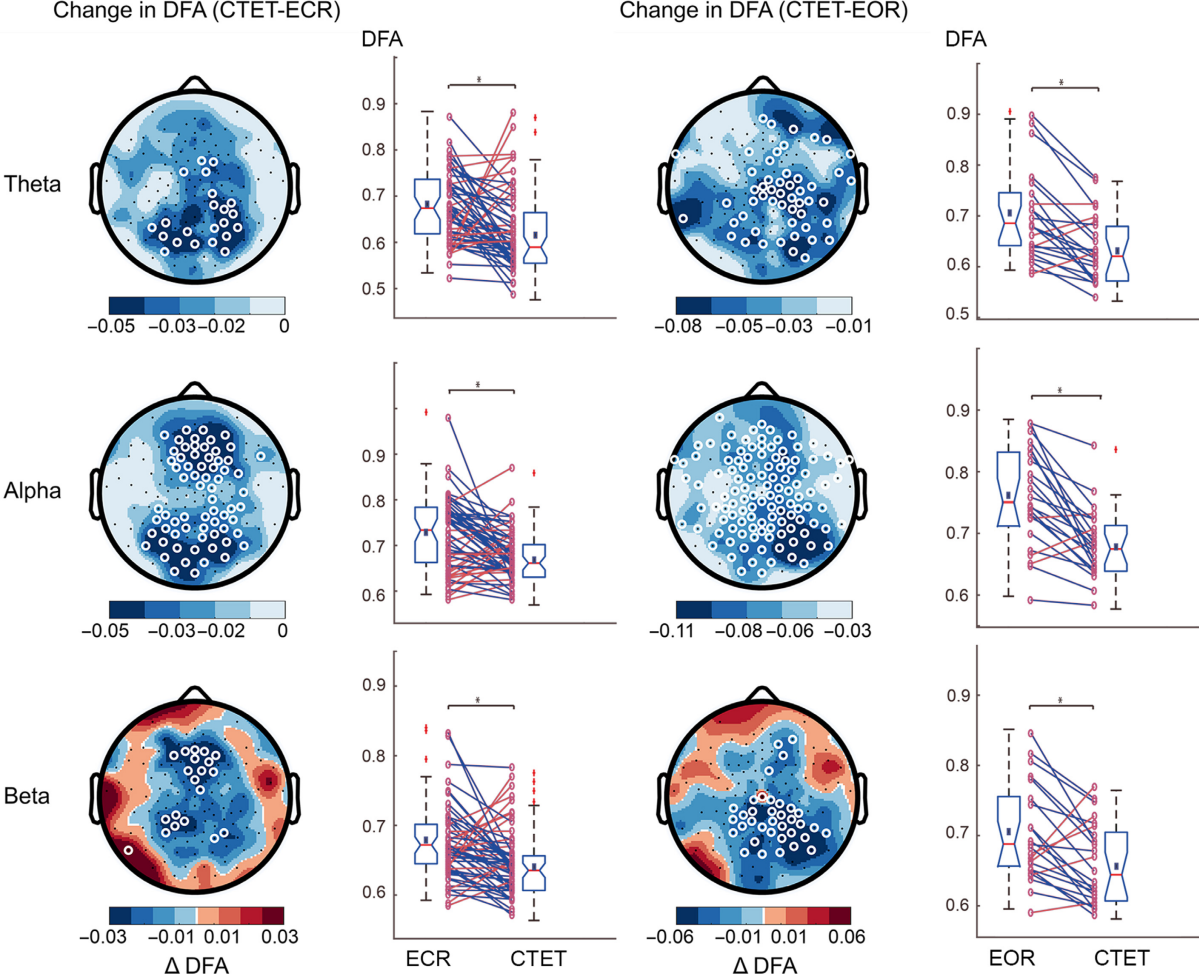
Strong reduction in long‐range temporal correlations (LRTC) during attention task compared to rest. Difference in detrended fluctuation analysis (DFA) exponents are shown in the left column for continuous temporal expectancy task (CTET) minus eyes‐closed rest (*n *=* *57), and in the right column for CTET minus eyes‐open rest (*n *=* *23). Individual‐subject exponent values averaged across significant electrodes are shown next to the topographic plots with lines connecting paired values. Subjects showing increases in LRTC are plotted in red, whereas decreases are plotted in blue. Following either rest condition, a widespread reduction in LRTC in the theta, alpha, and beta bands was found during CTET, especially in fronto‐parietal and occipital regions. White circles denote channels with *P *<* *0.05 (*t*‐test, multiple comparisons corrected).

Numerous studies have documented the presence of significant LRTC during eyes‐closed rest; however, little is known about the impact of focused attention on the temporal complexity of ongoing oscillations. Comparing the CTET and ECR conditions, we observed a widespread reduction in LRTC in the theta (α_ECR_ = 0.66 ± 0.01, α_CTET_ = 0.61 ± 0.01; t_56_ = −3.1, *P *=* *0.002), alpha (α_ECR_ = 0.71 ± 0.01, α_CTET_ = 0.67 ± 0.01; t_56_ = −3.5, *P *=* *0.001), and beta (α_ECR_ = 0.66 ± 0.01, α_CTET_ = 0.64 ± 0.01; t_56_ = −2.9, *P *=* *0.005) bands, especially at fronto‐parietal and occipital regions (Fig. 2). In a subset of 23 subjects, we had eyes‐open rest data available, which showed a similar effect, from EOR to CTET theta (α_EOR_ = 0.69 ± 0.02, α_CTET_ = 0.63 ± 0.01; t_22_ = −4.5, *P *= 0.0002), alpha (α_EOR_ = 0.75 ± 0.02, α_CTET_ = 0.68 ± 0.01; t_22_ = −5.2, *P *= 0.0003), beta (α_EOR_ = 0.70 ± 0.01 α_CTET_ = 0.66 ± 0.01; t_22_ = −3.1, *P *=* *0.0005) (Fig. 2). Thus, actively engaging in a sustained attention task is associated with reduced LRTC in occipito‐parietal and frontal scalp regions in the theta, alpha, and beta‐frequency bands relative to rest—whether resting with eyes open or closed.

### High sensorimotor LRTC during rest predict fast reaction times

**Figure 3:**
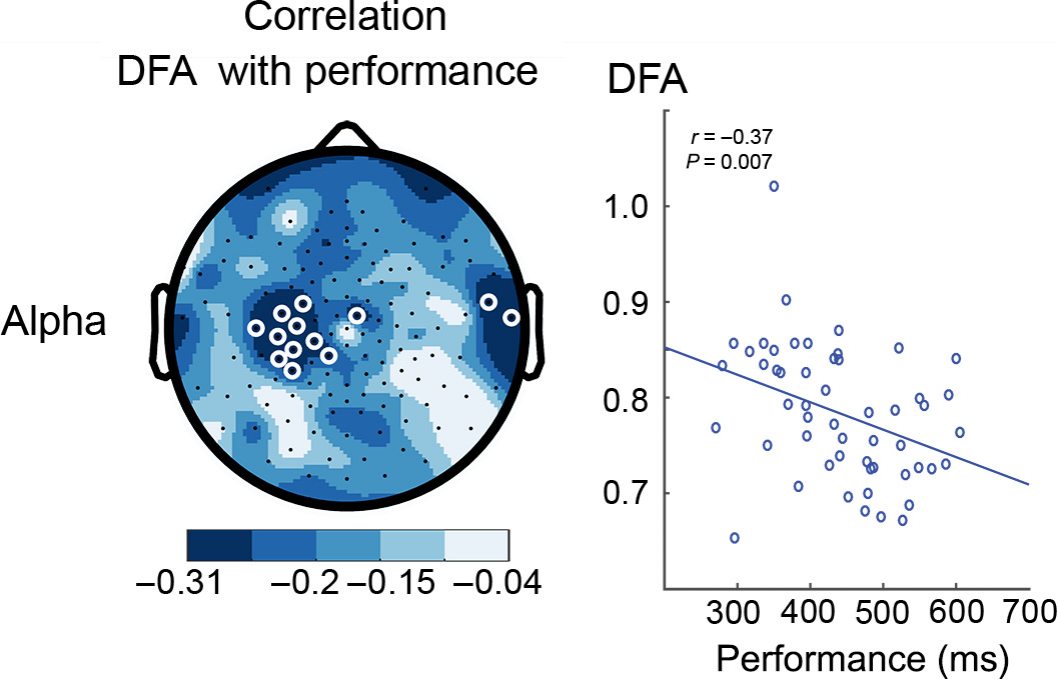
Long‐range temporal correlations (LRTCs) during eyes‐closed rest (ECR) predict reaction times in the subsequent attention task. Correlations between the reaction‐time performance and LRTC during the rest condition for the alpha oscillations indicate significant negative associations over the contralateral sensorimoter region (white circles denote channels with *P *<* *0.05, multiple comparisons corrected). Participants with high detrended fluctuation analysis (DFA) in brain activity during resting state showed better performance (faster reaction times) during the subsequent attention task.

Previous studies have found that resting‐state oscillatory dynamics is predictive of temporal fluctuations in perceptual (Palva *et al*., 2013) and motor task performance (Smit *et al*., 2013). To test whether resting‐state LRTCs can predict performance in a sustained visual attention task (see Methods and Fig. 1), we correlated the LRTC of oscillations during eyes‐closed rest with the reaction‐time performance in the CTET that required speeded reactions to deviant stimulus durations. Interestingly, high LRTC of resting‐state oscillations in the alpha band over the sensorimotor region—contralateral to the right hand used in the subsequent attention task—predicted better reaction‐time performance (*r*_*56*_ **= −0.37, *P *=* *0.007; Fig. 3). We did not observe significant associations between normalized oscillation power and reaction time for any of the frequency bands (data not shown).

### Good performance is associated with low LRTC and high spectral power during CTET

**Figure 4:**
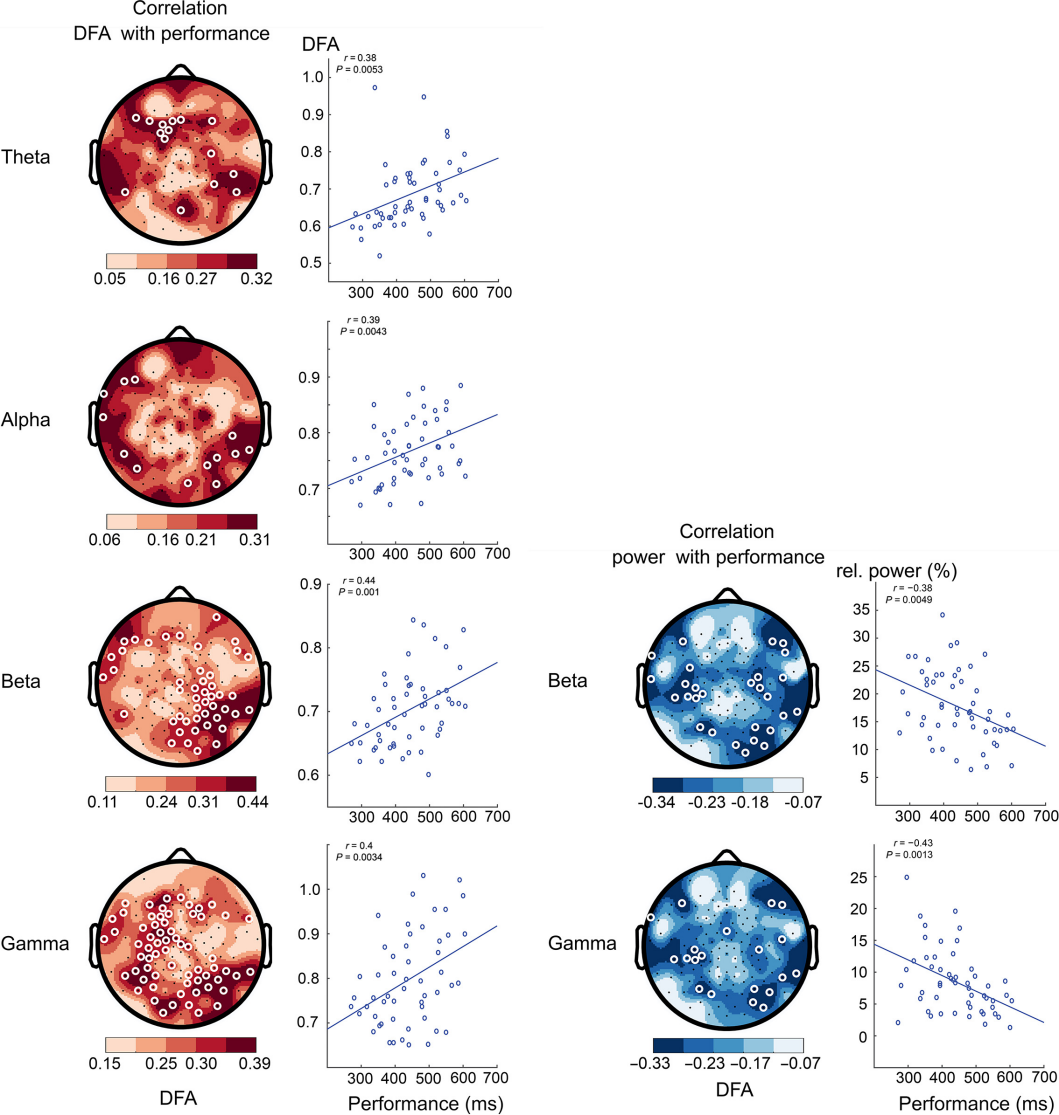
Low long‐range temporal correlations (LRTC) and high power in beta/gamma oscillations during high‐performance sustained visual attention. Left column: Correlation (Pearson's r) between LRTC during continuous temporal expectancy task (CTET) and reaction‐time performance in the theta, alpha, beta, and gamma frequency bands. Participants with lower detrended fluctuation analysis (DFA) in brain activity during the attention task showed faster reaction times. Right column: Correlation between the behavioral performance and the spectral power of the beta and gamma frequency bands (white circles denote channels with *P *<* *0.05, multiple comparisons corrected). Participants with higher power in brain activity during the attention task showed better performance (faster reaction times).

Next, we investigated the direct relationship between behavioral performance and brain oscillations during the CTET experiment. Positive correlations between LRTC and reaction times were most pronounced in occipital and parietal regions of the beta (*r*_*56*_ = 0.44, *P *=* *0.001) and gamma (*r*_*56*_ = 0.40, *P *=* *0.003) frequency bands (Fig. 4). For the theta (*r*_*56*_ = 0.38, *P *=* *0.005) and alpha (*r*_*56*_ = 0.39, *P *=* *0.004) oscillations, the correlations were also positive, with similar topographic distribution to that of the beta and gamma oscillations, but with fewer significant channels. Additionally, we found that traditional EEG spectral power in the beta (*r*_*56*_ = −0.38, *P *=* *0.005) and gamma (*r*_*56*_ = −0.43, *P *=* *0.001) frequency bands showed the opposite correlation with performance in similar regions to those that showed significant associations with LRTC (Fig. 4). Together, this indicates that focusing well and responding quickly to deviant image display times is facilitated by stable and strong high‐frequency oscillations.

### The change in LRTC from ECR to CTET also predicts performance

**Figure 5:**
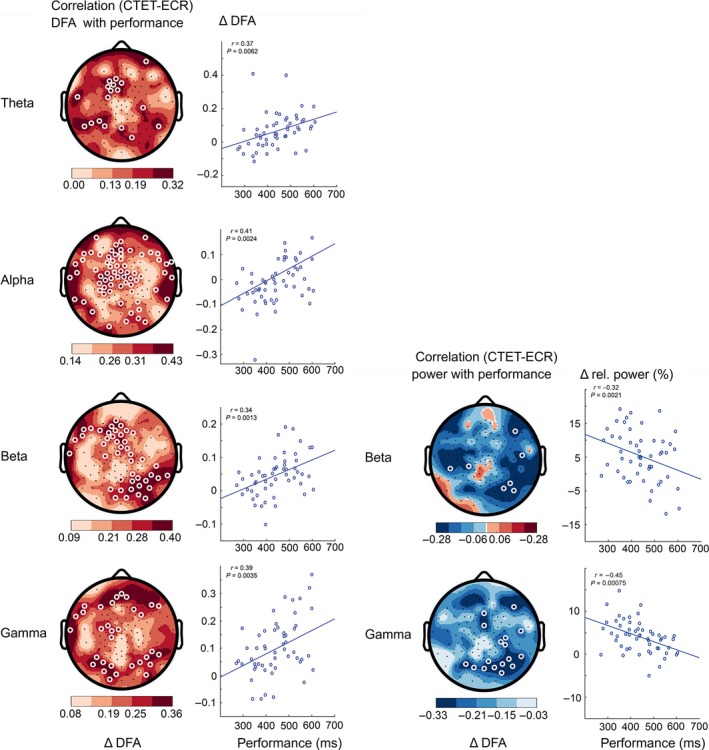
Suppressed long‐range temporal correlations (LRTC) and elevated power in beta/gamma oscillations during high‐performance sustained visual attention compared to rest. Left column: Correlation between the reaction‐time performance and the continuous temporal expectancy task (CTET) minus eyes‐closed rest (ECR) difference in detrended fluctuation analysis (DFA) exponents for theta, alpha, beta, and gamma frequency bands. Participants with little change or slight decrease in DFA during the attention task compared to the rest condition showed better performance. Right column: Oscillation power in beta and gamma bands shows the opposite pattern: Performance is best in participants with the strongest change in neuronal oscillation power from rest to the attention task (white circles denote channels with *P *<* *0.05, multiple comparisons corrected). Note: The correlation with the beta‐band power did not pass the binomial criterion, but the similarity with the topography of the association with LRTC of beta oscillations speaks for a valid trend. Here, the correlation with an occipital channel neighboring to O2 is shown.

Additional analysis revealed that not only *during* the CTET task there is a positive correlation of LRTC with performance, but also the change of LRTC from ECR to CTET that is correlated with reaction time in the theta (*r*_*56*_ = 0.37, *P *=* *0.006), alpha (*r*_*56*_ = 0.41, *P *=* *0.002), beta (*r*_*56*_ = 0.34, *P *=* *0.001), and gamma (*r*_*56*_ = 0.39, *P *=* *0.004) bands (Fig. 5). A reduction in LRTC from rest condition to CTET correlates with better performance, while an increase is associated with reduced performance. EEG spectral power only showed significant associations in the gamma band over occipital regions *(r*_*56*_ = −0.45, *P *=* *0.0007*)* and a trend in the beta band. This shows that not only the state of oscillatory properties *during* the task, but also changes and the direction of changes in LRTC from resting state to the attentional state that are related to performance.

## Discussion

In this study, we have tested the hypothesis that the temporal complexity of oscillations is related to behavioral performance during sustained attention. We have investigated the change in amplitude and LRTC of neuronal oscillations in a task requiring a single and sustained focus. We found that performance success during a sustained attention task was related to ongoing oscillations in the human EEG: Good performance in the behavioral task was related to higher spectral power and lower LRTC of beta and gamma oscillations especially in occipital regions. Interestingly, the opposite relationship between reaction times and LRTC was observed in the resting state: the higher the LRTC of alpha oscillations, the better the performance. Therefore, we showed that good performance is not only associated with the state of weak LRTC during the task (Fig. 4), but also to the ability to suppress an increase in LRTC relative to the individual resting‐state values (Fig. 5). This was especially clear in the beta and gamma bands in occipital and mid‐frontal regions. Specifically, we note that the positive associations in Fig. 5 relates to the poor performance of participants presenting with an increase in LRTC during the attention task compared to rest, which is the opposite association to what one would have expected if the correlations were caused by the statistical phenomenon of regression to the mean (Barnett *et al*., 2005). Taken together, our results suggest that the framework of critical brain dynamics is relevant for understanding mass‐neuronal mechanisms of sustained attention.

### Why would critical‐state dynamics be beneficial for attention?

The human attention system combines two seemingly opposing functional properties: the ability to stay focused for a sustained period of time and rapid switching in response to changing internal or external demands. This is conceptually similar to the properties of dynamical systems operating near a critical state in which meta‐stable patterns form and rapidly dissolve upon perturbations (Deco *et al*., 2013; Linkenkaer‐Hansen *et al*., 2001; Singer, 2013; Tognoli & Kelso, 2014). A system operating near criticality is poised between ordered (sub‐critical) and disordered (super‐critical) states, optimally combining the ability to form patterns while also responding swiftly to input. Indeed, critical‐state dynamics have been related to optimal information processing (Shew & Plenz, 2013) and capacity (Shew *et al*., 2009), high robustness against perturbations (Hahn *et al*., 2010), and largest dynamic range in sensory processing (Gautam *et al*., 2015; Kinouchi & Copelli, 2006). In a supercritical state, the brain would be in a state of hyperarousal, whereas in a subcritical state propagation of neuronal activation is suppressed. Empirical (Beggs & Plenz, 2003) and theoretical (Poil *et al*., 2012) studies have highlighted the importance of balanced excitation and inhibition for a network to exhibit critical dynamics, which may be an explanation for why scale‐free dynamics of oscillations is altered in many brain disorders, including depression (Linkenkaer‐Hansen *et al*., 2005); Alzheimer (Montez *et al*., 2009); epilepsy (Monto *et al*., 2007); Parkinson's (Hohlefeld *et al*., 2012), and autism (Lai *et al*., 2010). It could also be that the excitation–inhibition balance is altered during task engagement.

### Why would focus change the temporal structure of the attentional system?

We showed that during the attention task the brain shifts from complex resting‐state dynamics to a temporally more homogeneous state. We interpret this change in dynamics as a transition from a ‘default’ close‐to‐critical resting state optimized for a broad range of environmental and internal demands (Beggs & Plenz, 2003) to a state of reduced propagation of input but increased attentional stability. Therefore, our observation allows for the hypothesis that changes in cognitive control according to demand can be quantified as an ability to suppress LRTC of ongoing neuronal oscillations.

It needs to be stated that it is not known which exact DFA exponent corresponds to the critical state, especially not if different oscillations have different maximum DFA exponents. That said, a high DFA exponent will always be closer to the critical state than a lower. Even if a network is in a super‐critical state DFA exponents will exhibit an increase when the network moves closer to the critical state. Therefore, our results support the idea that on the one hand, a state close to criticality may be beneficial when versatility is required, whereas continued attentive visual processing is associated with a reduction in criticality fluctuations. Possibly due to successfully suppressing, the occurrence of non‐task‐related information propagation such as mind‐wandering episodes and the increased variability these would cause. Similarly, dynamical changes have also been shown in a transition from resting state to task activity in EEG during a cued response task (He *et al*., 2010) and fMRI BOLD activations (He, 2011), which in both cases were associated with a decrease in the long‐range memory of the signal. A reduction in exponents indicates less autocorrelations and, therefore, less influence on future dynamics (Eke *et al*., 2002; Mandelbrot & Van Ness, 1968), hence possibly less distractions from the focused task at hand, for example the mind wandering off the task. Further, it has been argued that less temporal redundancy leads to more efficiency in processing (He, 2011), perhaps enhancing the conscious experience of the object of focus, therefore possibly allowing for faster recognition of the deviant display time and subsequent quicker reaction.

Therefore, we believe part of the importance of our results relate to showing that critical‐state dynamics of oscillations are not *per se* beneficial for the performance of a given task, despite much of the interest in critical brain dynamics has been motivated by the superior computational properties of neuronal networks poised at the critical state. Default mode network alpha oscillations have previously been associated with attention to internal as opposed to sensory information and could also have influenced the current task (Carhart‐Harris *et al*., 2014). However, the occipital topographies of effects—and especially the increasing beta/gamma power jointly with a reduced complexity of the temporal structure of these oscillations—suggest that successfully sustaining attention is reflected in an uninterrupted processing of the visual stimuli.

### Importance of alpha oscillations during rest

The correlation of LRTC of resting‐state oscillations in alpha with reaction time was a noteworthy finding, because of its distinct sensorimotor region topography. Although we did not perform source modeling of oscillations, based on previous studies on alpha oscillations reactivity to finger movements (e.g., Fig. 2H in (Smit *et al*., 2013)) such as required in the present paradigm—it seems very likely that the correlation reflect sensorimotor oscillations. We see it as preliminary evidence that a motor region operating close to criticality is advantageous for a quick motor response, which warrants further investigation.

### Importance of beta and gamma oscillations

The strongest correlations with good performance were found in the beta and gamma frequency bands. Both frequency ranges have been reported for their significance in attentive processing of visual stimuli (Jensen *et al*., 2007; Wróbel, 2000) and conscious perception (Keil *et al*., 1999; Meador *et al*., 2002). Oscillatory phase synchronization has been reported as a mechanism for long‐distance neuronal communication (Salinas & Sejnowski, 2001) and facilitation of selective sensory gating (Jensen & Mazaheri, 2010). We indeed find that higher power in the beta and gamma band during the visual task is related to better performance. Interestingly, however, the temporal structure decreased in both frequency bands with better performance. Showing that next to increased power and synchronization, information propagation through temporal coding in the form of LRTC is also of significance.

### Observations are not confounded by signal‐to‐noise effects

A factor that could influence the DFA estimate is the signal‐to‐noise ratio of the signal. The lower this ratio, the more the estimated scaling is biased toward an uncorrelated noise signal (Linkenkaer‐Hansen *et al*., 2007). Intriguingly, we found that the DFA showed a negative correlation in relation to performance, while at similar scalp locations, the spectral power in the beta and gamma showed a positive correlation (Fig. 3). Thus, subjects presenting with high performance on the sustained visual attention task had the weakest LRTC and the highest power of beta and gamma oscillations. Therefore, the LRTC results cannot be accounted for by signal‐to‐noise ratio effects.

### Impact of period stimulation on LRTC

The majority of past studies investigating LRTC in neuronal oscillations have focused on resting‐state recordings (Hardstone *et al*., 2012). Periodic stimulation as used here, however, can introduce a characteristic scale in the amplitude modulation of oscillations, such as the mu rhythm during periodic stimulation of the median nerve (Linkenkaer‐Hansen *et al*., 2004a). Importantly, periodic modulation—whether in the form of a reduction or an enhancement of oscillation amplitudes—cannot in itself give rise to scale‐free modulation of oscillations. This has been studied previously, also by simulating periodic modulation of amplitudes by stereotypical stimulus responses (Linkenkaer‐Hansen *et al*., 2004b, b). We, therefore, consider it unlikely that event‐related potentials or modulation of neuronal oscillations could explain the associations between LRTC and performance reported here. We find it a more likely interpretation that steady focus of attention has a ‘whitening effect’ on neuronal dynamics, suppressing the complexity of fluctuations while trying to maintain a steady brain state, which is an observation we have also made in the absence of sensory stimulation while meditators perform focused attention meditation (Irrmischer *et al*., 2017).

### Observations are not due to the difference between eyes‐open test vs. eyes‐closed rest

To test whether changes in LRTC between CTET and ECR were dependent on the difference between eyes open vs. eyes closed, we did the control experiment in which we included an eyes‐open rest (EOR). Results showed a reduction in LRTC in virtually the same areas (Fig. 2). Therefore, we conclude that focused visual attention suppresses LRTC beyond that of the eyes‐open effect.

### Outlook

Our results suggest that the framework of critical brain dynamics is relevant for understanding mass‐neuronal mechanisms of sustained attention and reaction‐time performance.

## Conflict of interest

The authors declare no competing financial interests.

## Author contributions

Mona Irrmischer involved in conceptualizing, conducting, recording and processing data, analyzing, and writing. Francesca Sangiuliano Intra involved in recording and processing the data. Simon‐Shlomo Poil involved in analysis. Huibert D. Mansvelder performed supervision and writing. Klaus Linkenkaer‐Hansen involved in conceptualizing, analyzing, supervision, and writing.

## Data accessibility

Data are available on request by the authors.

**Abbreviations**
CTET: continuous temporal expectancy task
DFA: detrended fluctuation analysis
ECR: eyes‐closed rest
EEG: electroencephalography
EOR: eyes‐open rest
fMRI BOLD: blood‐oxygen level‐dependent functional magnetic resonance image
LRTC: long‐range temporal correlations
