# Supplementary material for: Strong long‐range temporal correlations of beta/gamma oscillations are associated with poor sustained visual attention performance
Source: Eur J Neurosci. 2017 Oct 6;48(8):2674–83. doi: 10.1111/ejn.13672 (PMC6221163; doi:10.1111/ejn.13672)
Supplement: Supplementary file 1 [file EJN-48-2674-s001.pdf]

European Journal of Neuroscience FENS Federation of European Neuroscience Societies

Strong long-range temporal correlations of beta/gamma oscillations are associated with poor sustained visual attention performance

Mona Irrmischer, Francesca Sangiuliano Intra, Huibert D. Mansvelder, Simon-Shlomo Poil and Klaus Linkenkaer-Hansen

Review timeline:

Submission date:	06 June 2017
Editorial Decision:	14 July 2017
Revision received:	27 July 2017
Accepted:	25 August 2017

Editor: Heleen Slagter

1st Editorial Decision 14 July 2017

Dear Mrs. Irrmischer,

Your manuscript was reviewed by two external reviewers as well as by the Section Editor, Dr. Heleen Slagter, and ourselves. The reviews collectively indicate that your work has generated new and important information. However, there are several substantial issues that need to be clarified/resolved before we can consider your manuscript further for publication in EJN. There is confusion about what your “lapse” trials represent and you will need to do a better job of describing your methods and analyses. There are also concerns raised about both noise contamination and potential contributions from the evoked potential to the oscillatory measures you report. You will need to make clear how these contributions can be accounted for and ruled out. Please reconsider your title along the lines suggested by Reviewer #1. Please also make sure to state that ethical approval was obtained and the name of the board that did so.

If you are able to respond fully to the points raised, we would be pleased to receive a revision of your paper within 12 weeks.

Thank you for submitting your work to EJN.

Kind regards,

John Foxe & Paul Bolam
Editors in Chief, EJN

Reviews:

Reviewer: 1 (Redmond O'Connell, Trinity College Dublin, Ireland)

Comments to the Author
In this paper the authors examine the extent to which long range temporal correlations (LRTC) in EEG oscillatory activity reflects task engagement during visual sustained attention. Participants completed a variant of the Continuous Temporal Expectancy Task, originally used in O'Connell et al (2009) and a resting recording session. The authors found significant correlations between RT and LRTC in multiple EEG bands.

I think this is an interesting paper and a novel approach to the analysis of EEG data that potentially sheds new light on the neural signatures of sustained attention. I do have some significant concerns about some aspects of the analysis that would need to be addressed.

Major Comments

1. It is stated that 'attention lapses were included in the reaction times in the form of time penalties'. It is not at all clear what this means. Presumably an attention lapse is a trial on which the participant failed to respond to a target? If so, then what time penalty was added? Is it that miss trials are assigned the longest

possible response time? If this was the procedure that was followed then it would require some further justification in my view. Assigning the longest RT to misses seems an arbitrary approach. Why not simply analyse LRTC as a function of hit/miss rate as well as RT on hit trials? What leaves me uncertain that I am correctly understanding the analysis is the assertion that trials with RT greater than 900ms were classified as misses. This implies that if a participant responded at 1000ms then this was counted as a miss which again seems entirely arbitrary.

2. To what extent can we be sure that the LRTC results are unaffected by SNR? I get that LRTC and absolute power go in opposite directions in the case of beta but they go in the same direction in the case of gamma. Did the authors try covaring or partialling out power in their analyses to fully exclude this potential confound?

3. The authors make the strong claim that these oscillatory LRTC results reflect critical cortical states but to what extent can we be sure that they are not driven by stimulus-evoked potentials which might serve to attenuate LRTC in certain bands while enhancing it in others. I note that in this case I do not have a clear sense of how ERPs might contribute to the present results but at minimum the authors should consider the issue in their Discussion and moderate their interpretation of the Results accordingly.

Minor Comments

When describing the CTET in the first instance it should be made clear that this paradigm was originally reported in O'Connell et al 2009 (note there is a typo and the citation is currently Connell et al).

'Cleaning procedure EEG', I suggest 'EEG pre-processing' as an alternative

'Sharp artifacts were manually cut out.' It should be possible for another researcher to replicate the methods that were used here so further information is required. How did the authors classify 'sharp artifacts'. The EEG segments were 10seconds long so how many segments were lost at this stage of the analysis?

I think the title could be improved. The reference to Critical-state Dynamics is a little strong given the evidence provided in this paper, I would suggest swapping this for 'Long term correlations in oscillatory activity' which is just as interesting a findings in my view and more defensible. Also I think the meaning of 'high performance' will be unclear to most readers (it's unclear to me at least!) and not necessary to get the message across

Reviewer: 2 (Christopher Kello, University of California Merced, USA)

Comments to the Author
The authors present an EEG study of LRTC in alpha, beta, and gamma bands (delta was measured but no results were reported in this band), and their relation to sustained attention. The authors find that 1) LRTC is stronger during rest than task performance, 2) LRTC during rest is positively related to performance, and 3) LRTC during test is negatively related to performance. The results are interesting and make a worthwhile contribution to the literature, but I had some questions about ambiguities, missing info, and theoretical interpretation. My general sense was that the results were more complicated than the conclusions, and the analyses were more exploratory than they appeared.

"A key prediction derived from this hypothesis, is that long-range temporal correlations in human EEG oscillations are suppressed during sustained attention and are related to behavioral performance."

"Comparing the CTET and ECR conditions, we observed a strong reduction in LRTC in the theta…" The effects are highly reliable, but the "strength" of an effect is typically refers to its effect size, and effect sizes are not reported. Looking at the effect means, the effect sizes may not be that strong. The authors should either report effect sizes or clarify that they are highly reliable rather than strong.

"Interestingly, high LRTC of resting-state oscillations in the alpha band over the sensorimotor region—contralateral to the right hand used in the subsequent attention task—predicted better reaction-time performance…" This result is interesting, but needs more context information: How well can the effect be localized, what do prior studies indicate about the region(s) that shows the effect, and how many other correlations were tested that did not come out reliable? Also, given that this region and band was not explicitly targeted, and many correlations were checked, how was the family-wise error rate taken into account?

The authors make a strong distinction between the change in DFA exponents from rest to task performance, and the effect of the DFA exponent values themselves during each phase of the experiment. However, it seems like these may not be distinct effects. For instance, if a DFA exponent is relatively high during rest, the DFA exponent is likely to be lower during task performance for purely statistical reasons, and thereby

EJN European Journal of Neuroscience FENS Federation of European Neuroscience Societies

result in a negative change. The authors should test whether the observed change effects are somehow above and beyond the effects of DFA exponent within the rest phase and within the task performance phase.

The authors theorize in terms of the critical state, but it is not made clear what DFA exponent corresponds with the critical state. A decrease in DFA exponent is a move away from the critical state only if the exponent starts out at or below critical. If it starts out above critical, then a decrease is actually a move towards the critical state. So, interpreting DFA exponents in terms of criticality requires a specific exponent associated with criticality.

In terms of criticality as a theoretical approach to interpreting the results, it was not clear why some results were found in alpha, and others in beta and gamma. In terms of criticality, when should a DFA exponent effect relate to performance and behavior, and when should it not?

Authors' Response 17 July 2017

Dear Heleen Slagter, John Foxe and Paul Bolam,

We would like to thank you for the timely and careful reviewer feedback, as well as the opportunity to revise and re-submit the manuscript. We believe the constructive comments made by the reviewers have helped us strengthen the paper considerably.

Major changes include:

- A new and improved title
- Improved rationale behind reaction time calculation (p. 7)
- We also discuss effects of signal-to-noise ratio on the estimates of long-range temporal correlations in neuronal oscillations and on the core findings (p. 13)
- Potential contributions from the evoked potential to the oscillatory measures (p. 13)

We believe that our revisions and additional analyses, including a new figure, adequately address the reviewers' concerns. Our point-by-point responses to their comments, with indications of how we have revised the manuscript, are included below.

Yours sincerely, on behalf of the co-authors,

Mona Irrmischer

Reviewer: 1

Major Comments

1. It is stated that 'attention lapses were included in the reaction times in the form of time penalties'. It is not at all clear what this means. Presumably an attention lapse is a trial on which the participant failed to respond to a target? If so, then what time penalty was added? Is it that miss trials are assigned the longest possible response time? If this was the procedure that was followed then it would require some further justification in my view. Assigning the longest RT to misses seems an arbitrary approach. Why not simply analyse LRTC as a function of hit/miss rate as well as RT on hit trials? What leaves me uncertain that I am correctly understanding the analysis is the assertion that trials with RT greater than 900ms were classified as misses. This implies that if a participant responded at 1000ms then this was counted as a miss which again seems entirely arbitrary.

The reviewer is correct that a recorded reaction time of 1000 ms was considered a miss and allocated a 900 ms reaction time when calculating the mean across all trials to prevent incorporating false responses to the following stimuli. Importantly, similar to the correlation with the reaction time, we observed a positive correlation between the number of errors and LRTC during CTET in the same frequency bands in similar scalp regions: alpha (r = .44, p = .0006), beta (r =.44, p = .0006), gamma (r =.41, p = .002). In the manuscript, we chose the combined reaction time as a more comprehensive measure of performance (1) because the effects were very similar and (2) to prevent an explosion in the number of figures. We added this in the manuscript in the methods (p. 7):

- "The observed reaction-time averages were calculated from the point in time when the target stimulus was displayed longer than non-target stimuli. The reaction time, therefore, includes both the time needed to notice the deviant and the time to react. The next stimulus is displayed 600 ms after and to prevent that wrong presses to non-target stimuli would count as a very slow reaction to the target stimulus, we defined the maximum allowed reaction time to 900 ms. To avoid short reaction times in subjects responding very fast but also missing several trials, and to obtain a comprehensive performance measure that also included the misses and too slow responses, we defined misses to have the longest reaction time allowed (i.e., 900 ms). We note that reaction times and number of errors exhibited very similar associations with LRTC of neuronal oscillations during CTET and, therefore, we only report the results for the reaction-time measure defined above."

2. To what extent can we be sure that the LRTC results are unaffected by S-N-R? I get that LRTC and absolute power go in opposite directions in the case of beta but they go in the same direction in the case of gamma. Did the authors try covaring or partialling out power in their analyses to fully exclude this potential confound?

The main results cannot be explained as an SNR effect so there was no reason to partial out power. We now explain this in the discussion (p. 13):

- *"Correlations between LRTC and performance are not confounded by signal-to-noise effects.* A factor that could influence the DFA estimate is the signal-to-noise ratio of the signal. The lower this ratio, the more the estimated scaling is biased towards an uncorrelated noise signal (Linkenkaer-Hansen et al., 2007). Intriguingly, we found that the DFA showed a negative correlation in relation to performance, whereas the spectral power in the beta and gamma bands showed a positive correlation in similar scalp locations (Figure 3). Thus, subjects presenting with high performance on the sustained visual attention task had the weakest LRTC and the highest power of beta and gamma oscillations. In other words, the correlations between LRTC and performance cannot be accounted for by signal-to-noise ratio effects."

3. The authors make the strong claim that these oscillatory LRTC results reflect critical cortical states but to what extent can we be sure that they are not driven by stimulus-evoked potentials which might serve to attenuate LRTC in certain bands while enhancing it in others. I note that in this case I do not have a clear sense of how ERPs might contribute to the present results but at minimum the authors should consider the issue in their Discussion and moderate their interpretation of the Results accordingly.

We now elaborate on the possibility of stimulus-evoked changes in LRTC in the discussion (p. 13):

- "*Impact of periodic stimulation on LRTC*. The majority of past studies investigating LRTC in neuronal oscillations have focused on resting-state recordings (Hardstone et al., 2012). Periodic stimulation as used here, however, can introduce a characteristic scale in the amplitude modulation of oscillations, as it has been shown for the mu rhythm during periodic stimulation of the median nerve (Linkenkaer-Hansen et al., 2004). Importantly, periodic modulation—whether in the form of a reduction or an enhancement of oscillation amplitudes—cannot in itself give rise to scale-free modulation of oscillations. This has been studied previously, also by simulating periodic modulation of amplitudes by stereotypical stimulus responses (Linkenkaer-Hansen et al., 2004). We, therefore, consider it unlikely that event-related potentials or modulation of neuronal oscillations could explain the associations between LRTC and performance reported here. We find it a more likely

interpretation that a steady focus of attention has a “whitening effect” on neuronal dynamics, suppressing the complexity of fluctuations while trying to maintain a steady brain state—something we have observed also in the absence of sensory stimulation in the context of experienced meditators performing focused attention meditation (Irrmischer et al., 2017).“

Minor Comments

When describing the CTET in the first instance it should be made clear that this paradigm was originally reported in O'Connell et al 2009

We apologize for the oversight, which was unintended. We did cite O'Connell et al. 2009; however, we now credit this important work in multiple places, including the legend to Figure 1, which shows how we adapted the original paradigm of (O’Connell et al., 2009).

'Cleaning procedure EEG', I suggest 'EEG pre-processing' as an alternative

Done.

'Sharp artifacts were manually cut out.' It should be possible for another researcher to replicate the methods that were used here so further information is required. How did the authors classify 'sharp artifacts'. The EEG segments were 10 seconds long so how many segments were lost at this stage of the analysis?

We clarified the methods (p.7):

- “All signals were visually inspected in windows of 10 seconds and transient artifacts, e.g., caused by head movements or eye blinks were manually marked and omitted from the subsequent computations of spectral power and DFA exponents. Typically, only 1–2 seconds around an artifact was marked.”

I think the title could be improved. The reference to Critical-state Dynamics is a little strong given the evidence provided in this paper, I would suggest swapping this for 'Long term correlations in oscillatory activity' which is just as interesting a findings in my view and more defensible. Also I think the meaning of 'high performance' will be unclear to most readers (it's unclear to me at least!) and not necessary to get the message across

The title now is:

- “Strong long-range temporal correlations of beta/gamma oscillations are associated with poor sustained visual attention performance”

Please also make sure to state that ethical approval was obtained and the name of the board that did so.

We have added a sentence to reference our ethical approval (see, p. 5).

- “All participants signed the informed consent, the protocol was approved by the The Scientific and Ethical Review Board (VCWE) of the Faculty of Psychology and Education, VU University Amsterdam.”

Reviewer: 2

My general sense was that the results were more complicated than the conclusions, and the analyses were more exploratory than they appeared.
“A key prediction derived from this hypothesis, is that long-range temporal correlations in human EEG oscillations are suppressed during sustained attention and are related to behavioral performance.”

We had this expectation due to results obtained in a study comparing focused attention meditation with rest. In that study, we observed a suppression of LRTC in experienced practitioners but not in novices (manuscript under review, reported at LM symposium: Irrmischer et al., 2017)*. We now added the citation in reference list.

And in the introduction on p. 4 as

- “The transition from resting-state to attention-task activity on the other hand shows a decrease in the long-range memory of the signal as found during focused attention meditation in experienced meditators (Irrmischer et al., 2017)….”

  and (p. 4):
- “In this paper, based on the earlier findings of reduced LRTC during focused attention meditation (Irrmischer et al., 2017), we propose the working hypothesis that the human brain is poised near a critical state that makes attention inherently unstable and, consequently, a less volatile brain state is desired when sustained focus of attention is required. A key prediction derived from this hypothesis, is that long-range temporal correlations in human EEG oscillations are suppressed during sustained attention and that such suppression may be related to behavioral performance.”

Additionally, statements and interpretations in the manuscript were adjusted to be more tentative. For example (Discussion, p. 12):

- Original: “According to the hypothesis that a focused attention system operates further away from the critical state than during less restricted tasks, such as resting with eyes closed, we also showed that during the attention task the brain shifts from complex resting-state dynamics to a temporally more homogeneous state.”

Was changed into:

- “We show that during the attention task the brain shifts from complex resting-state dynamics to a temporally more homogeneous state characterized by weaker LRTC”

“Comparing the CTET and ECR conditions, we observed a strong reduction in LRTC in the theta…” The effects are highly reliable, but the “strength” of an effect is typically refers to its effect size, and effect sizes are not reported. Looking at the effect means, the effect sizes may not be that strong. The authors should either report effect sizes or clarify that they are highly reliable rather than strong.

We thank the reviewer for pointing out this inaccurate formulation. We meant:

- “we observed a widespread reduction in LRTC”.

We also updated the legend to Figure 2. Furthermore, to illustrate the reliability of the effects, we expanded Figure 2 to show individual-subject effects.

EJN European Journal of Neuroscience FENS Federation of European Neuroscience Societies

We feel that this illustration efficiently conveys the robustness of the effects; however, we did calculate the effect sizes, which were in the medium range for the condition CTET-ECR (theta: d = 0.55, r = .27, alpha: d = .55 r = .27 and beta: d = .47, r = .23), and medium to high in the CTET-EOR condition (theta: d = 0.76, r = 0.354, alpha: d = 1.04, r = .46, beta: d = .62, r = .3).

"Interestingly, high in the alpha band over the sensorimotor region—contralateral to the right hand used in the subsequent attention task—predicted better reaction-time performance…" This result is interesting, but needs more context information: How well can the effect be localized, what do prior studies indicate about the region(s) that shows the effect, and how many other correlations were tested that did not come out reliable? Also, given that this region and band was not explicitly targeted, and many correlations were checked, how was the family-wise error rate taken into account?

The correlation of LRTC of resting-state oscillations in alpha with reaction time was indeed an interesting finding especially as neuronal dynamics of resting-state alpha-band oscillations recorded at precentral sites were found to predict scaling exponents of tapping behavior (Smit et al., 2013). We did not correct for similar comparisons in other frequency bands; however, we see it as preliminary evidence that a motor region operating close to criticality is advantageous for performance, which warrants further investigation, and therefore worth reporting.

We have added a note on this to the discussion on p. 13:

- "*Importance of alpha oscillations during rest*. The correlation of LRTC of resting-state oscillations in alpha with reaction time was a noteworthy finding, because of its sensorimotor region. Although we did not perform source modeling of oscillations, based on previous studies on alpha oscillations reactivity to finger movements (e.g., Figure 2H in (Smit et al., 2013)) such as required in the present paradigm—it seems very likely that the correlation reflect sensorimotor oscillations. We see it as preliminary evidence that a motor region operating close to criticality is advantageous for a quick motor response, which warrants further investigation."

The authors make a strong distinction between the change in DFA exponents from rest to task performance, and the effect of the DFA exponent values themselves during each phase of the experiment. However, it seems like these may not be distinct effects. For instance, if a DFA exponent is relatively high during rest, the DFA exponent is likely to be lower during task performance for purely statistical reasons, and thereby result in a negative change. The authors should test whether the observed change effects are somehow above and beyond the effects of DFA exponent within the rest phase and within the task performance phase.

In case the reviewer refers to the 'regression towards the mean' effect, the assumption is that the values of the first (or second) measurement are extreme and that the second (or first) measure is closer to the mean (Barnett et al., 2005). In our data both cases are well within the physiological norm of exponents falling in the range of 0.6–0.9 (Smit et al., 2011; Poil et al., 2012). The direction of change in the DFA exponent between task and rest is also consistent across subjects (Fig 2), meaning it is unlikely that these DFA exponents are fluctuating randomly around a 'mean value'. Importantly, because of the positive correlation (Figure 4), one would have expected a negative correlation between change in LRTC from ECR to CTET and task performance, whereas we observed a positive association (Fig. 5). We now address this in the discussion (p. 11).

- "Finally, we showed that good performance is not only associated with the state of weak LRTC during the task (Fig. 4), but also to the ability to suppress an increase in LRTC relative to the individual resting-state values (Fig. 5). This was especially clear in the beta and gamma bands in occipital and mid-frontal regions. Specifically, we note that the positive associations in Figure 5 relates to the poor performance of participants presenting with an increase in LRTC during the attention task compared to rest, which is the opposite association to what one would have expected if the correlations were caused by the statistical phenomenon of regression to the mean (Barnett et al., 2005)."

Additionally, we also checked that the mean of the CTET condition actually falls within the standard deviation of the ECR (EOR), therefore also decreasing the likelihood that the first measure was extremely different from the second:

[ECR: theta (α_{ECR}=0.66 STD: 0.8, α_{CTET}=0.61 STD: 0.09), alpha α_{ECR}=0.71 STD: 0.08, α_{CTET}=0.67 STD: 0.05), and beta (α_{ECR}=0.66 STD: 0.05, α_{CTET}=0.64 STD: 0.05) , EOR: theta (α_{ECR}=0.69 STD: 0.09, α_{CTET}=0.63 STD: 0.07), alpha (α_{ECR}=0.75 STD: 0.08, α_{CTET}=0.68 STD: 0.06), beta (α_{ECR}=0.70 STD: 0.07 α_{CTET}=0.66 STD: 0.06)]

The authors theorize in terms of the critical state, but it is not made clear what DFA exponent corresponds with the critical state. A decrease in DFA exponent is a move away from the critical state only if the exponent starts out at or below critical. If it starts out above critical, then a decrease is actually a move towards the critical state. So, interpreting DFA exponents in terms of criticality requires a specific exponent associated with criticality.

The reviewer raises an important question regarding which DFA exponent corresponds to the critical state. In particular, it is not known whether neuronal networks poised at criticality result in different DFA exponents for different oscillations. That said, a high DFA exponent should always be closer to the critical state than a lower. Thus, it is not entirely correct that a network in a super-critical state will exhibit decreasing DFA exponents when the network moves closer to the critical state. We have explained this relationship more carefully on p. 13:

- "We note that the exact DFA exponent corresponding to neuronal networks in a critical state is not known, especially not whether different oscillations have different maximum DFA exponents. Nonetheless, one would always expect DFA exponents to reach their maximum value when the networks generating the oscillations operate in the critical state (Poil et al., 2012). Thus, even when a network is in a super-critical state DFA exponents will exhibit an increase when the network moves closer to the critical state."

We explain the DFA exponents in the methods only in terms of LRTC (Methods, p. 8)

- "A DFA exponent $\alpha = 0.5$ indicates randomly fluctuating oscillation amplitudes (no temporal structure), whereas $0.5 < \alpha < 1.0$ indicates LRTC with the temporal inhomogeneity of fluctuations increasing with increasing DFA exponents."

And the critical state also only in terms of LRTC (Abstract, p. 2)

- E.g.: "Such long-range temporal correlations (LRTC) are thought to reflect neuronal systems poised near a critical state"

We do not give an exact DFA value for the 'critical state' in the brain but deliberately work in terms of relative distance to the critical state, because we know that the DFA exponent will always be highest when the networks produce neuronal avalanches with critical exponents ($k\sim1$) and, thus, are critical (Poil et al., 2012). Moving away from this peak point leads to a decrease in DFA (<1).

In terms of criticality as a theoretical approach to interpreting the results, it was not clear why some results were found in alpha, and others in beta and gamma. In terms of criticality, when should a DFA exponent effect relate to performance and behavior, and when should it not?

We agree with the reviewer that this is an interesting question; however, it is also a very broad one that we cannot exhaustively answer with our data.

We have added a note on this to the Discussion (p. 13).

"Therefore we believe part of the importance of our results relate to showing that critical-state dynamics of oscillations are not *per se* beneficial for the performance of a given task, despite much of the interest in critical brain dynamics has been motivated by the superior computational properties of neuronal networks poised at the critical state. Default mode network alpha oscillations have previously been associated with attention to internal as opposed to sensory information and could also have influenced the current task (Carhart-Harris *et al.*, 2014). However, the occipital topographies of effects—and especially the increasing beta/gamma power jointly with a reduced complexity of the temporal structure of these oscillations—suggest that successfully sustaining attention is reflected in an uninterrupted processing of the visual stimuli."

References

Barnett AG, van der Pols JC, Dobson AJ (2005) Regression to the mean: What it is and how to deal with it. Int J Epidemiol 34:215–220.

Carhart-Harris RL, Leech R, Hellyer PJ, Shanahan M, Feilding A, Tagliazucchi E, Chialvo DR, Nutt D (2014) The entropic brain: a theory of conscious states informed by neuroimaging research with psychedelic drugs. Front Hum Neurosci 8:20 Available at: http://www.pubmedcentral.nih.gov/articlerender.fcgi?artid=3909994&tool=pmcentrez&rendertype=abstract.

Hardstone R, Poil SS, Schiavone G, Jansen R, Nikulin V V, Mansvelder HD, Linkenkaer-Hansen K (2012) Detrended fluctuation analysis: A scale-free view on neuronal oscillations. Front Physiol 3 NOV.

*Irrmischer, M., Houtman, S.J., Mansvelder, H.D., Tremmel, M., Ott, U., Linkenkaer-Hansen, K, F Meditation reduces complex amplitude fluctuations of neuronal oscillations. Landelijk Mindfulness Symposium, Leiden, 19 May, 2017. Abstract: https://www.radboudcentrumvoormindfulness.nl/wp-content/uploads/2016/02/Focused-attention-meditation-EEG-results-Mona-Irmischer.pdf

Linkenkaer-Hansen K, Nikulin V V., Palva JM, Kaila K, Ilmoniemi RJ (2004) Stimulus-induced change in long-range temporal correlations and scaling behaviour of sensorimotor oscillations. Eur J Neurosci 19:203–211.

Linkenkaer-Hansen K, Smit DJA, Barkil A, Van Beijsterveldt TEM, Brussaard AB, Boomsma DI, Van Ooyen A, De Geus EJC (2007) Genetic Contributions to Long-Range Temporal Correlations in Ongoing Oscillations. J Neurosci 27:13882–13889.

O'Connell RG, Dockree PM, Robertson IH, Bellgrove MA, Foxe JJ, Kelly SP (2009) Uncovering the Neural Signature of Lapsing Attention: Electrophysiological Signals Predict Errors up to 20 s before They Occur. J Neurosci 29:8604–8611 Available at: http://www.ncbi.nlm.nih.gov/pubmed/19571151%5Cnhttp://www.jneurosci.org/cgi/doi/10.1523/JNEUROSCI.5967-08.2009%5Cnpapers3://publication/doi/10.1523/JNEUROSCI.5967-08.2009.

Poil S-S, Hardstone R, Mansvelder HD, Linkenkaer-Hansen K (2012) Critical-state dynamics of avalanches and oscillations jointly emerge from balanced excitation/inhibition in neuronal networks. J Neurosci 32:9817–9823 Available at: http://www.jneurosci.org/content/32/29/9817.short.

Smit DJA, de Geus EJC, van de Nieuwenhuijzen ME, van Beijsterveldt CEM, van Baal GCM, Mansvelder HD, Boomsma DI, Linkenkaer-Hansen K (2011) Scale-free modulation of resting-state neuronal oscillations reflects prolonged brain maturation in humans. J Neurosci 31:13128–13136.

Smit DJA, Linkenkaer-Hansen K, Geus EJC de (2013) Long-Range Temporal Correlations in Resting-State Alpha Oscillations Predict Human Timing-Error Dynamics. J Neurosci 33:11212–11220 Available at: http://www.jneurosci.org/content/33/27/11212.
